# The Effects of Flexibility on dsDNA–dsDNA Interactions

**DOI:** 10.3390/life12050699

**Published:** 2022-05-07

**Authors:** Chuanying Chen, B. Montgomery Pettitt

**Affiliations:** Department of Biochemistry and Molecular Biology, Sealy Center for Structural Biology and Molecular Biophysics, University of Texas Medical Branch, Galveston, TX 77555, USA; ch2chen@utmb.edu

**Keywords:** molecular dynamics simulation (MD), potential of mean force (PMF), dsDNA–dsDNA interactions, flexibility

## Abstract

A detailed understanding of the physical mechanism of ion-mediated dsDNA interactions is important in biological functions such as DNA packaging and homologous pairing. We report the potential of mean force (PMF) or the effective solvent mediated interactions between two parallel identical dsDNAs as a function of interhelical separation in 0.15 M NaCl solution. Here, we study the influence of flexibility of dsDNAs on the effective interactions by comparing PMFs between rigid models and flexible ones. The role of flexibility of dsDNA pairs in their association is elucidated by studying the energetic properties of Na^+^ ions as well as the fluctuations of ions around dsDNAs. The introduction of flexibility of dsDNAs softens the vdW contact wall and induces more counterion fluctuations around dsDNAs. In addition, flexibility facilitates the Na^+^ ions dynamics affecting their distribution. The results quantify the extent of attraction influenced by dsDNA flexibility and further emphasize the importance of non-continuum solvation approaches.

## 1. Introduction

It has been shown experimentally [1,2,3] that ion-mediated attraction happens between like-charged polyelectrolytes such as dsDNA. The attraction is not captured by the well-known Poisson–Boltzmann (PB) theory and hence other approaches have been proposed. An extension of condensation theory [4] on two infinite line charges in a highly dilute 1:1 salt solution proposed that the attraction comes from the increase of translational entropy of condensed counterions. As two dsDNAs approach in an intermediate region, the volume of the condensation region increases. Another possible explanation of attractive interactions is counterion correlation, which can be explained by two mechanisms. One [5] involves counterions that reposition themselves and form a strongly correlated liquid on the surface of dsDNAs (similar to a Wigner lattice) due to strong interactions with polyelectrolytes, and with each other. The other explanation [6,7] considers the attractions that originate from charge fluctuations along the rods. The movement of condensed counterions introduces the fluctuations of the local charges on monomers along the polymer, resulting in dipole–dipole attractions and even higher-order multipole interactions. In addition, a more structural argument posits that an ideal alignment of dsDNA pairs produces an “electrostatic zipper” [8], in which positive counterions in the grooves of one dsDNA can interact with the negatively charged phosphate groups of the neighboring dsDNA. This idea also demonstrates a helical-specific recognition of dsDNA–dsDNA interactions.

In previous work, we found that the localization of ions near charged groups can give rise to a local energy minimum at an optimal short separation due to the formation of a hydrogen bond (HB) network among Na^+^ ions, water and phosphate atoms of dsDNAs [9]. The studies mentioned above are mostly based on rigid bodies. Although the rigid structures used were relaxed [10] to improve the theoretical model, possible physical mechanisms responsible for the attraction are complicated and collectively influenced by the spatial distributions of counterions, solvent and dsDNAs.

Interplay between DNA flexibility and the surrounding counterions has been studied experimentally and theoretically [11,12,13,14,15]. A long dsDNA is a semi-flexible polyelectrolyte with persistence length. Its overall flexibility can be roughly described by a worm-like chain model (WLC) [16,17] in which the chain is relatively rigid at a small length scale but turns flexible over a longer length. Its rigidity is balanced by electrostatic effects (like-charge repulsion) and various elastic effects (e.g., base pair stacking), and strongly depends on the counterion types, valence, shape and concentration [14,18]. The counterion density and fluctuations can reduce the chain persistence length and could lead to instability of rodlike chain conformations [19]. For a short dsDNA fragment it is arguable among the experimental, theoretical and simulation studies that the duplex is much softer than predicted by a WLC model [20,21,22]. Thus, there exists flexibility in a short dsDNA fragment, which in turn influences the distributions of counterions and could lead to differences in correlations between counterions and DNA and among counterions themselves.

In this paper, we present a comparison, using oriented flexible structures of identical dsDNA models, of the potential of mean force (PMF) of the pairing processes along the interhelical separation. We wish to consider the effects of local dsDNA flexibility on parallel strands and investigate the radial and azimuthal angular dependence of the forces. The PMF calculations were carried out utilizing the adaptive biasing force (ABF) method [23,24] sampled via molecular dynamics (MD) simulations. The simulations sampled forces between dsDNA pairs in three different helical alignments in 0.15 M NaCl salt solution. Although the ion-mediated interaction of infinitely dilute dsDNA pairs is thermodynamically unfavorable in this monovalent salt solution, an attractive component exists at short surface separation (within 8 Å of contact), which comes from the collective correlations of the counterions and water and the relative helical alignment of dsDNA pairs [9]. We investigated the effects of dsDNA flexibility on dsDNA–dsDNA interactions considering the surrounding counterions, particularly by studying energetic and dynamic behaviors of Na^+^ ions around the dsDNAs. The calculations provide the extent of attraction influenced by dsDNA flexibility when compared to rigid models and further emphasize the importance of counterion correlations that are not captured in continuum PB theory.

## 2. Methods

### 2.1. MD Simulations

**Figure 1:**
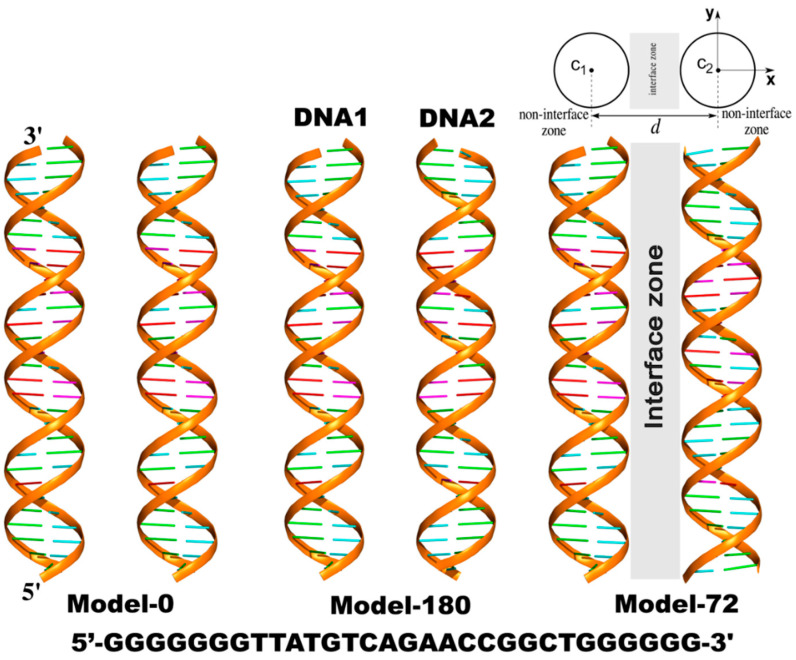
Three models of parallel 30-bp dsDNA pairs in the simulations. The helical axis is along the z-axis. From the top view of the systems, c_1_(*c*_1*x*_,*c*_1*y*_,*c*_1*z*_) and c_2_(*c*_2*x*_,*c*_2*y*_,*c*_2*z*_) represent the helical centers of DNA1 and DNA2, respectively. *d* is the interhelical distance. Any point (*x*,*y*,*z*) is considered inside dsDNA when its radial distance from the corresponding c_1_ or c_2_ is smaller than the radius of the dsDNA, R_DNA_ = 10 Å. Non-interface zone includes each cylindrical region of dsDNA extending to the bulk solution (*x* > c_1*x*_ or *x* < c_2*x*_).

Three models of dsDNA pairs having different helical alignments were considered (Figure 1). The first one (Model-0) has two identical sequences of 30-base-pair DNAs (DNA1 and DNA2) parallel to each other sampled at various interhelical separations, *d*. The helical axis is along the z-axis. In the other two models, DNA2 was rotated about its helical axis by 72 degrees (Model-72) and 180 degrees (Model-180), respectively.

Each model was then solvated in a TIP3P [25] water box with dimensions of 99 × 99 × 103 Å. Na^+^ and Cl^−^ ions were randomly added to both neutralize the system and set the salt concentration at 0.15 M. The resulting system contained about 101,000 atoms. Each DNA duplex is effectively infinite as each DNA duplex strand is covalently bonded to itself through the periodic boundary.

MD simulations were performed using NAMD 2.14 software [26] with the CHARMM36 force field parameter set [27]. NBFIX corrections [28] were applied to Lennard–Jones interaction potentials between Na^+^ ions and Cl^−^ ions and phosphate oxygens. Such corrections are used to fit to osmotic pressure for concentrated aqueous solutions or confined systems [28,29]. Particle mesh Ewald [30] was used to calculate long-range electrostatic interactions, and van der Waals interactions were truncated at 12 Å. All bonds were constrained using the SETTLE algorithm [31] and equations of motion were integrated with a time step of 2 fs. Temperature was controlled with Langevin coupling with a damping coefficient of 1/ps. After 50K steps of energy minimization, the system was heated up from 0 K to 310 K with restraints on the DNAs with a force constant of 500 kcal/(mol∙Å^2^). The system was then switched to the NVT ensemble for equilibration for over 100 ns until the salt and solvent distributions were stable.

### 2.2. ABF Calculations

We calculated the PMFs on the three geometric models using two DNA flexibility settings: rigid body and flexible structures, resulting in a total of six systems. The PMF calculations were carried out utilizing the ABF method implemented in the Colvars module [24] of NAMD. In the ABF calculation, the orientations of the dsDNAs were constrained. DNA1 was fixed in its global position, and DNA2 was permitted to diffuse along the reaction coordinate of interhelical distance *d* by a force that is adjusted via ABF in response to effective energetic barriers. The biasing force acting on DNA2 is equal in magnitude and opposite in sign to the mean force on DNA2. Instantaneous values of the force were accumulated in bins of 0.1 Å along *d* from 31 to 21 Å center to center. To enhance sampling of the distribution of configurations and increase the efficiency of the calculations, the PMF pathway was divided into three consecutive, non-overlapping windows. 

The ABF calculations started at 31.0 Å for both flexible and rigid models sharing the same structures. Then different collective variable restraints were introduced by means of harmonic potentials with corresponding force constants, k: (i) positional restraints of DNA1 with k = 80 kcal/(mol∙Å^2^), (ii) rotational restraints of DNA1 and DNA2 with k = 2000 kcal/(mol∙deg^2^); (iii) positional restraint of DNA2 to restrict its movement in the yz-plane, k = 80 kcal/(mol∙Å^2^). Those restraints allow the dsDNA atoms to be locally flexible and yet maintain their separation and orientation features. For the rigid body systems, we introduced strong restraints on root-mean-square deviation (RMSD) of both dsDNAs with k = 2000 kcal/(mol∙Å^2^).

The PMF uncertainty was estimated using the method proposed by Henin and Chipot [23]. The PMF was considered converged once the distribution of the instantaneous force at each bin followed a Gaussian distribution and sampling was reasonably uniform along the reaction coordinate in each window (Figure S1). For each system the estimate of the sampling time required to complete the entire calculation is ~400 ns.

### 2.3. Differential Entropy from Density Fluctuations

To investigate possible ion correlations, we estimated the entropy change associated with the flexibility influence on the statistics of sodium ion density fluctuations. Theoretically, fluctuations of water/ions density in small microscopic volumes obey Gaussian statistics [32,33]. Following the coarse-grained density field method [34,35], we can compute the density field at a series of spatial grid points **r** at time *t*:

(1) $$\bar{\rho \left(r,t\right)}=\underset{i}{\sum }\phi \left(\left|r-r_{i}\left(t\right)\right|;\xi \right)$$

and

(2) $$\phi (r)={\left(2\pi \xi^{2}\right)}^{-d/2}exp\left(-r^{2}/2\xi^{2}\right)$$

where *r* is the distance of the *i*th particle of interest (Na^+^ ion here) from **r**, *ξ* is the Gaussian width and chosen to be 3.0 Å, and *d* is dimensionality. The sum is over all Na^+^ ions in the whole space. 

For Gaussian density functions, entropy has an analytic form proportional to the determinant of the covariance matrix. We estimate the correlation of multivariate Gaussians, which can be quantified by a differential entropy *S*(*x*) [36,37],

(3) $$S\left(x\right)=-\int_{-\infty }^{+\infty }p\left(x\right)\ln\left[p\left(x\right)\right]dx$$

where *x* is a random variable with an expected value of *μ* and a continuous density function *p*(*x*). *p*(*x*) is a multivariate Gaussian density function given by

(4) $$p\left(x\right)=\frac{1}{{\left(2\pi \right)}^{N/2}{\left|\sum \right|}^{1/2}}exp\left[-\frac{1}{2}{\left(x-\mu \right)}^{T}\Sigma^{-1}\left(x-\mu \right)\right]$$

in which Σ is the covariance matrix. In this study, $\Sigma_{ij}=\;\delta \rho_{i}\delta \rho_{j}={\left(\rho -\bar{\rho }\right)}_{i}{\left(\rho -\bar{\rho }\right)}_{j}$ and $\bar{\rho_{i}}$ is the time averaged density of Na^+^ ions at point *i*. Then the final entropy can be deduced from

(5) $$S=const+\frac{k_{B}}{2}\;\ln\left(\left|\Sigma \right|\right)$$

where |Σ| is the determinant of covariance matrix, and *k_B_* is the Boltzmann constant. The constant term in Equation (5) depends on grid spacing and the number of grid points studied. Under the same conditions, we estimate the difference of entropy, ∆*S* = *S*(*flexible*) − *S*(*rigid*), from density fluctuations between the rigid and flexible systems.

### 2.4. Localization of Diffusion Coefficients of Na^+^ Ions

The local diffusion constant was calculated by using a finite difference expression [38,39] with a grid spacing of 1.0 Å.

(6) $$6D_{uvw}=\frac{1}{t_{2}-t_{1}}<|{\left(r\left(t_{2\;}\right)-r\left(t_{0}\right)\right)}^{2}-{\left(r\left(t_{1}\right)-r\left(t_{0}\right)\right)}^{2}|>$$

where *r*(*t*_0_) is the initial position at instant *t*_0_; *D_uvw_* is the local diffusion coefficient at a grid point *uvw* and was computed whenever |*r*(*t*_0_) *− r_uvw_*| < 1.0 Å; *t*_1_ and *t*_2_ were fixed at 1 ps and 2 ps, respectively, assuming the diffusion regime would be reached after 1 ps [40] and would not diffuse beyond 3 Å from *r*(*t*_0_).

## 3. Results and Discussion

### 3.1. Potential of Mean Force

**Figure 2:**
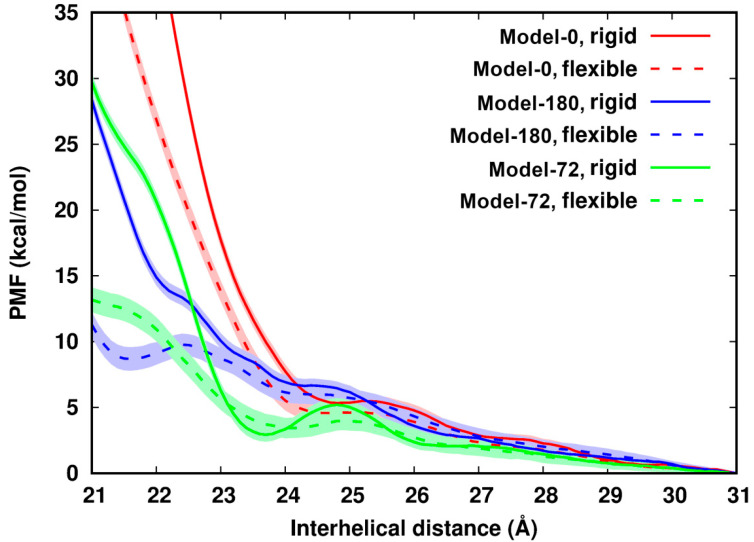
Potential of mean force of pairing two identical dsDNAs with difference helical features along the interhelical distance. The uncertainty (in shade) is up to 1.0 kcal/mol. The curves are arbitrarily set to zero at 31 Å.

In Figure 2, we observe that for all six systems their PMF profiles along *d* deviate from the continuum picture or PB theory. The PMF profiles can be divided into three regions: (i) in the region of *d* > 26 Å, the PMFs cannot be distinguished, which reflects the formally dominating long-ranged repulsive electrostatic interactions between two like-charged polyelectrolytes; (ii) in a range of ~24 Å < *d* < 26 Å, the PMFs establish a plateau or local minimum as has been seen before [9], an indication of the existence of attractive components of the interactions; (iii) in a region of *d* < ~24 Å, the PMF profiles display remarkable differences depending on the helical alignments of dsDNA pairs. The values of the PMFs in Model-72 and Model-180 are much lower than those in Model-0. This further demonstrates specific recognition of dsDNA–dsDNA in term of helical alignment [8,9,41]. 

Comparison of rigid and flexible systems shows that dsDNA flexibility softens the contact wall of the PMFs of dsDNA pairing in all three models. The influence from flexibility becomes apparent when two dsDNAs get close. In Model-72 as an example: from *d* = 23 Å to *d* = 22.0 Å, the difference of the PMFs between the flexible to the rigid system increases by 15 times from 0.64 kcal/mol to 9.7 kcal/mol, many times larger than thermal energy. In addition, we observed that in the distance range between 23.5 Å and 25.5 Å, the rigid Model-72 forms an energy barrier of about 2.3 kcal/mol. DNA flexibility lowers the energy barrier by as much as 1.3 kcal/mol and flattens the PMF curve.

We wish to investigate how flexibility enhances the pairing process. We might posit that the enhancement could be contributed by positive configurational entropy of dsDNA. Due to the constraint on dsDNAs to remain a helical arrangement in this study, during the whole simulation the RMSDs of all atoms from corresponding initial structures for all flexible systems are ~1.0 Å, much smaller than 3~9 Å which was inferred from an experiment [42] for dsDNAs in free motion. The RMS fluctuations (RMSF) of base groups are about 0.48~0.71 Å, 0.72~0.84 Å for sugar groups, and 0.85~1.2 Å for phosphate groups, respectively. The RMSFs are all slightly smaller than the corresponding groups of dsDNA in free motion [43]. Both RMSDs and RMSFs of the dsDNAs in our model systems are independent of distance spacing, so the configurational entropy contribution is not considered in this study.

To further investigate influences of flexibility on dsDNA–dsDNA pairing, we chose ten configurations at several distance separations and extended MD simulations for 18 ns more. The trajectories were saved every 0.1 ps for analysis. According to the PMF profiles, we chose 22.4 Å (a local maximum) and 24.4 Å (in the plateau) for Model-180; 22.4 Å, 23.7 Å (a local minimum for the rigid system and in the plateau for the flexible system), 24.9 Å (in a plateau) for Model-72.

### 3.2. vdW Interactions

**Figure 3:**
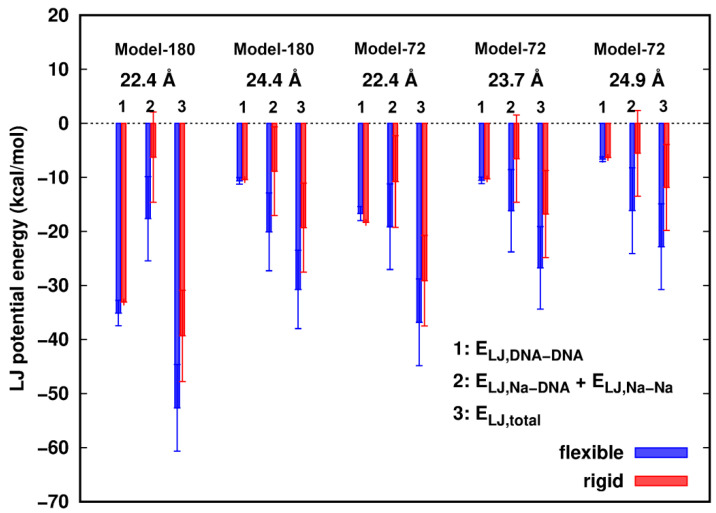
Lennard–Jones (LJ) potential energies of Na^+^ ions participated dsDNA–dsDNA interactions. Error bars represent standard deviations.

When surface separation between two dsDNAs is as short as ~2.4 Å, smaller than the diameter of a water molecule, we investigated the role of the vdW interactions in the dsDNAs pairing process. The vdW interaction was modeled by the Lennard–Jones (LJ) potential [27]. The differences of LJ potential energies of water with dsDNAs between the rigid and flexible structures in all models were estimated to be less than 1.0 kcal/mol, so we mainly focus on the dsDNA–dsDNA interactions with the participation of counterions. In Figure 3, we observed that the LJ energies of two dsDNAs, E_LJ,DNA-DNA_, show little difference between the rigid and flexible systems. In addition, the total LJ energies, E_LJ,total_, in all cases are attractive, and are more favorable in the flexible structures than in the rigid ones. The total energy difference, ∆E_LJ,total_ = E_LJ,total_(flexible) − E_LJ,total_(rigid), is mainly contributed by the interactions of the Na^+^ ions with the dsDNAs. In the rigid models, in events where Na^+^ ions are moving in close proximity of dsDNA atoms, vdW clash could happen, which would result in more repulsion between the Na^+^ ions and dsDNAs. In contrast, flexibility provides sufficient relaxation of the structures and allows the vdW contact wall to shift and consequently reduce the clashes of counterions with the DNA atoms.

Although the total LJ component is attractive for the approach of two dsDNAs with each other, the total interactions are energetically unfavorable, indicating that the electrostatic components are dominantly repulsive between isolated dsDNAs given the surrounding ions and solvent.

### 3.3. Ion Distributions and Electrostatic Energy of Ions around dsDNA Pairs

A previous study [9] revealed that close proximity of dsDNAs to each other has essentially no influence on the fraction of charge inside the grooves. The charge fraction around one dsDNA is larger than the prediction of Manning counterion condensation theory [44,45] for a single dsDNA. The large value of charge fraction here is due to the combination of Na^+^ ions correlated with the dsDNA, and the Na^+^ ion atmosphere shared with the neighboring dsDNA.

Considering the ion mobility near condensed dsDNA molecules and the heterogeneous environment of dsDNA, we discretized the space into a three-dimensional grid with a spacing of 1.0 Å. At a voxel we counted the number density of Na^+^ ions and calculated the electrostatic energy of this Na^+^ ion with the dsDNAs and the other Na^+^ ions using the standard Ewald summation [46,47]. In this way, the electrostatic energy contains information on local variations of Na^+^ ions and local correlations among the Na^+^ ions and the dsDNA charges.

From the number density we calculated the radial distribution of the Na^+^ ions from the helical center of the dsDNAs. For the non-interface zone (Figure 1), to avoid mutual interference in the interface from each dsDNA, we restricted the grid points to each dsDNA hemicylinder extending into the bulk solution. For the interface zone between two dsDNAs, a grid point was assigned to DNA1 when its x-coordinate is less than the x-coordinate of the midpoint of both dsDNA centers; otherwise it was assigned to DNA2. Radial number density distributions of Na^+^ ions in the interface and non-interface zone were calculated by averaging densities within the cylindrical shells from the center of each dsDNA with a layer spacing of 0.5 Å.

**Figure 4:**
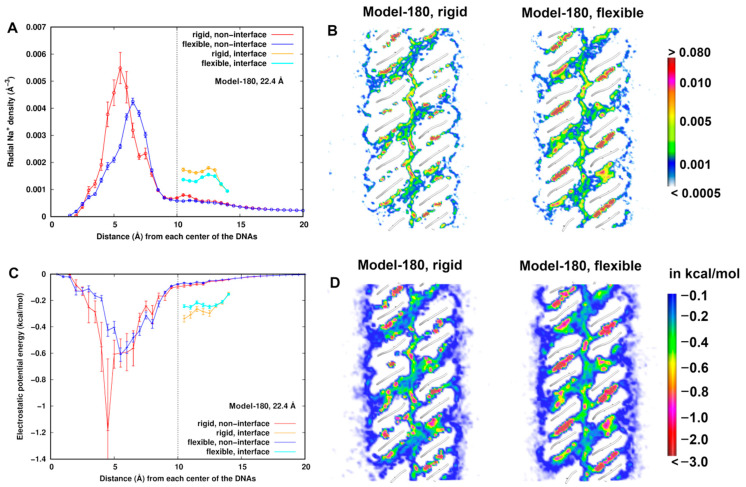
At *d* = 22.4 Å for Model 180, (**A**) radial number density distribution of Na^+^ ions from the helical center of the dsDNAs. (**B**) Comparison of number densities of Na^+^ ions between the flexible and rigid system. The number density is in the unit of Å^−3^. (**C**) Radial distribution of electrostatic energy of Na^+^ ions from the helical center of the dsDNAs. (**D**) Comparison of electrostatic potential energy of Na^+^ ions with the dsDNA pairs between the flexible and rigid system. A cross section (x–z plane) is displayed.

Figure 4A,C displays the radial distributions of Na^+^ ions number density and electrostatic energy, respectively, for Model-180 with *d* = 22.4 Å as an example. We observed that the density profile is correlated to the electrostatic energy profile as expected, particularly within 6 Å from the helical center of the dsDNAs, where a higher density is found in the rigid model than in the flexible one. The electrostatic energy reaches a minimum value at ~4.5 Å but with a large uncertainty. Possible explanations of such difference are that (i) due to flexibility, the electrostatic potentials at some local regions are averaged, resulting in the change of the electrostatic environment of the dsDNAs. In the rigid model, the sodium ions are likely to have stronger interactions with dsDNAs in some limited locations. In particular, they could have higher probabilities of direct contact with dsDNA (Figure S2) with a long lifetime. Direct contacts give rise to more favorable electrostatic energy at short range. Alternatively, (ii) dsDNA flexibility induces higher mobility of sodium ions. Even though direct contacts are possible, the densities are averaged in a fixed shell to yield a lower density distribution.

A radial distribution describes an average quantity in space, so we further compared the number density and electrostatic energy of sodium ions at cross sections between the rigid and flexible structures (Figure 4B,D). In both structures, the lowest electrostatic energy regions around dsDNA are found in the grooves and interface region. These lower electrostatic energies correspond to higher sodium densities in the corresponding regions. In addition, the distribution is more structured along the minor groove of the flexible systems, indicating flexibility of dsDNAs indeed influences the dynamic averaging of the Na^+^ ions. When the mobility is relatively low, Na^+^ ions are concentrated in a local region with a long lifetime. When the mobility is relatively high (e.g., in the minor groove) in the flexible model, the densities or electrostatic energies in a local region are smeared with a short lifetime. The density and electrostatic energy distribution profiles of Na^+^ ions in Model-72 are similar and displayed in Figure S3 in the SI text.

The rigid systems have higher Na^+^ ion densities and electrostatic potential energies inside dsDNAs within 6 Å from the helical axis and in the interface zone, which classically would indicate more screening of the dsDNA charges. We see the flexibility at short range dominating these screening effects. In addition, ∆E_LJ,total_ are similar at about −10 kcal/mol in all configurations studied. Thus, there must be other influences from flexibility when *d* < 23 Å. We found that the electrostatic energy distribution shows non-mean field interactions of Na^+^ ions with dsDNAs, which has implications for ion fluctuations and different dynamic behaviors of ions.

### 3.4. Differential Entropy from Density Fluctuations

Counterions could exhibit correlations in fluctuations in distinct local volumes. Due to the different spatial limits around the two dsDNAs, we focus on three regions: the minor groove, major groove and interface. Applying a coarse-grained procedure to each of the three regions with the collection of $\delta \rho \left(r\right)=\left(\rho \left(r\right)-\bar{\rho \left(r\right)}\right)$ and producing the observed correlation of $\delta \rho_{i}\delta \rho_{j}$, we calculated the entropy change of density fluctuations of the ions near flexible structures relative to the rigid ones. If Na^+^ ions are restricted in the rigid model, we expect low fluctuations and a smaller magnitude of entropy as well. For all configurations, we used the same grid spacing of 1.0 Å and for the configurations having the same relative helical angle we restricted the same number of grid points in the minor and major groove region, respectively. As a result, the constants involved in *S* (Equation (5)) are canceled in calculations of the entropy difference ∆*S*.

**Figure 5:**
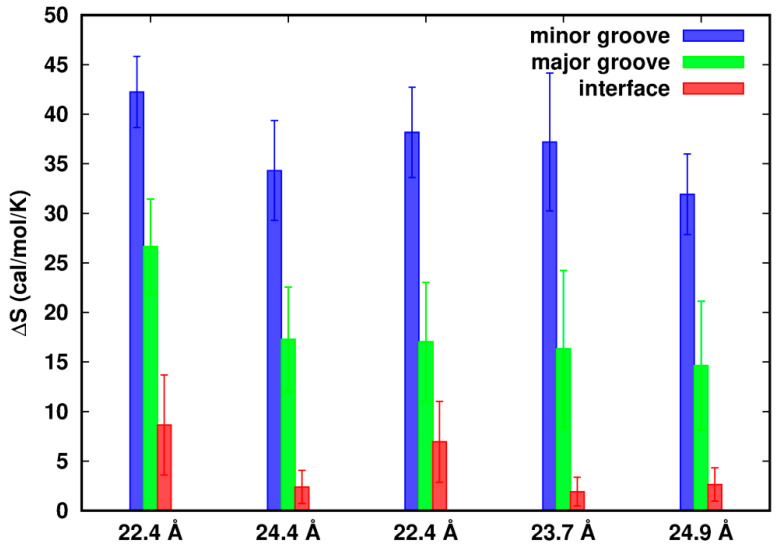
Difference of entropy from density fluctuations of the flexible systems relative to the rigid ones. The error bars represent the standard errors.

In Figure 5, we observe that in all cases the values of ∆*S* in the three regions are positive, indicating higher density fluctuations in the flexible systems. In a confined volume close to the dsDNAs, flexibility of the dsDNAs confers movements to the Na^+^ ions, resulting in relatively large fluctuations in density. Positive entropy also suggests a favorable free energy contribution to the interactions of dsDNA–dsDNA.

Although we restrict the study of entropy to the limited spaces, we cannot exclude possible correlations among these regions and the dsDNAs. Ha and Liu [6] showed that an increase of charge fluctuation of DNAs not only helps screen the electrostatic repulsion but also helps contribute to the dipole, quadrupoles and even higher-order multipoles along the DNAs. These multipoles can interact attractively with other multipoles (or monopoles), either on the same rod or on neighboring rods. Assuming there were little ion correlations, ∆*S* in the minor groove or major groove would be the same along *d* as well as in the interface zone sharing the same number of grids. However, we observed that for Model-180, ∆*S* in the minor groove/major groove at *d* = 22.4 Å is more positive than that at *d* = 24.4 Å. Similarly for Model-72, ∆*S* in the interface zone at *d* = 22.4 Å is more positive than that at *d* = 23.7 Å, indicating that ion fluctuations and correlations are strongly influenced by the close proximity of two dsDNAs.

### 3.5. Localization of Diffusive Dynamics of Na^+^ Ions

**Figure 6:**
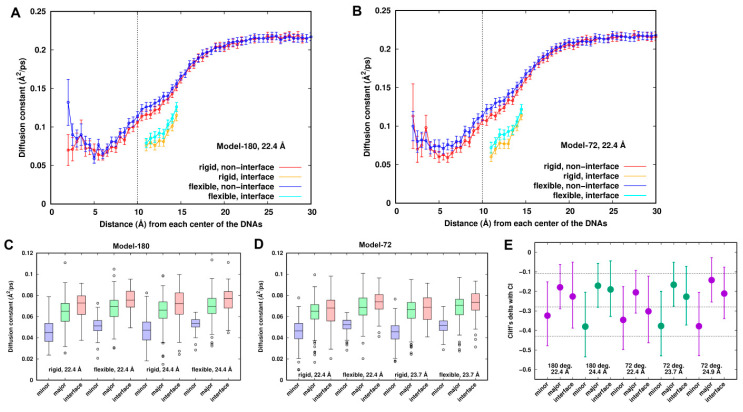
Comparison of local diffusion constants of sodium ions between the rigid and flexible systems. (**A**,**B**) Radial distribution of diffusion constants in the interface and non-interface zone for Model-180 and Model-72 at 22.4 Å, respectively. (**C**,**D**) Boxplots of local diffusion coefficients of Na^+^ ions around the DNA pairs for Model-180 and Model-72. (**E**) Cliff’s delta effective sizes with the error bars of 95% confidence interval. An effect size of +1.0 or −1.0 indicates the absence of overlap between the two groups, whereas 0.0 indicates that group distributions overlap completely. Generally, Cliff’s delta effect sizes of 0.11, 0.28 and 0.43 correspond to small, medium and large effects, respectively, displayed in the dash line.

As suggested above, “smeared” density and electrostatic energy in the minor groove implies high mobility of the Na^+^ ions. So, we studied the mobility of Na^+^ ions by localizing diffusion coefficients of the Na^+^ ions around the dsDNAs. The radial distribution of the diffusion coefficient of the Na^+^ ions (Figure 6A,B) shows that the calculated diffusion coefficient in the bulk is 0.21 ± 0.01 Å^2^/ps. This is slightly higher than another simulated result (Na^+^ ions: 0.17 Å^2^/ps [38]) and the experimental data (0.12 Å^2^/ps [48]). It could be an artifact of the force field or using a damping coefficient of 1.0 ps^−1^ in temperature control in the simulation. The lowest spatially localized averaged diffusion constants are identified with the values of ~0.06 Å^2^/ps at around ~5.5 Å, which are mainly located in the minor groove. The Na^+^ ions in the interface are less diffusive compared to those in the other similar shell distances when mediating the interactions between the two dsDNAs.

Figure 6A,B also shows that the Na^+^ ions are slightly more mobile around the DNAs in the flexible systems than in the rigid ones for all the systems, suggesting different dynamic behaviors of Na^+^ ions between the rigid and flexible systems. Diffusion coefficients were categorized into three groups, the minor groove, major groove and interface of the dsDNAs. To remove the noise due to low populations at some locations, we divided the 18-ns trajectory into nine blocks, calculated the diffusion coefficient at each grid point for each block, extracted the points which are ~70% overlapped, and finally smoothed the points by weighted averaging of the six closest neighbors until the separation of the sites was larger than 2.8 Å. Such a method has been successfully applied to identify hydration sites and effective sodium sites around proteins and DNA [9,49].

Comparison between the rigid and flexible systems is displayed in Figure 6C,D for Model-180 and Model-72, respectively. The mobilities of the Na^+^ ions increase in the order of minor groove < major groove ≤ interface for all systems, reflecting the different dynamic behaviors around the dsDNAs. It can be inferred from the figures that the datasets have a non-normal distribution and contain some extreme values. To compare the diffusion coefficients between the rigid and flexible systems, we performed Wilcoxon rank sum test [50,51] with the null hypotheses being equal distribution of the rigid and flexible systems at the 5% level of significance. Along with the *p*-value of the test, we estimated the effective size that describes the magnitude of the difference. We used Cliff’s delta [52], which is the probability that a value from one group (e.g., rigid system) is greater than a value from the other (e.g., flexible system) group, minus the reverse probability.

Figure 6E depicts a representation of Cliff’s delta effect sizes and their 95% confidence interval. The *p*-values from Wilcoxon rank sum test are all less than the significance level of 0.05. Thus, we can conclude that diffusion coefficients in the rigid systems are significantly different from those in the flexible systems. For the minor groove, the effect size is medium, and there is a ~70% chance (Table S1) that a location randomly chosen from the flexible systems has a higher diffusion coefficient than a location randomly chosen from the rigid systems. Such a chance is lower for both the major groove and the interface, having a value of ~60% with a small effect size.

In addition to spatial heterogeneity of the Na^+^ ions in terms of diffusion coefficients, we investigated the extent of the timescale during which the Na^+^ ions can reside in a local position or site. Localized residence times around the dsDNAs are displayed in Figure S4. Local residence times decrease in the order of minor groove > major groove ≥ interface region. The decreasing order is consistent with the increasing order of local diffusion coefficients. Almost all the sites having residence time longer than 1 ns are in the minor groove of the rigid systems. At those sites, the Na^+^ ions are partially dehydrated to directly contact nucleobase groups and/or O4’ of the sugar groups, and they are also in strong contacts indirectly via water with the DNAs. While in the similar locations in the flexible system, the residence times shrink to a few tens or hundreds of pico-seconds. In the interface zone, the majority of residence times become shorter, around 10~30 ps, in the flexible systems, while they are 20~50 ps in the rigid systems.

## 4. Conclusions

In this paper we focus on the influence of flexibility on dsDNA–dsDNA interactions by comparing rigid systems with flexible ones in the dsDNA pairing process in 0.15 M NaCl solution. The calculated PMF curves along the interhelical distance between two parallel dsDNAs indicate that flexibility enhances the dsDNA pairing process, particularly in a short distance region (*d* < 23 Å). Local flexibility of a molecule easily allows sub-Angstrom displacements in response to strong perturbing forces, such as the electrostatic field and vdW sphere contact of other surrounding molecular atoms. Thus, flexibility affects not only energetic properties of Na^+^ ions but also dynamic behaviors associated with close motion around dsDNAs, which in turn will affect dsDNA–dsDNA interactions.

It has been proposed that local alignment and pairing of dsDNAs in a “protein-free” environment is an initial step in homologous recombination [53,54,55,56,57]. Sequence-dependent attractive interactions are governed by local attractive interactions. A mutual electrostatic complementarity model [58] was provided to interpret the mechanism of homologous pairing. However, the calculations were electrostatically mean-field in a continuum solvent using torsionally rigid DNA, and so may be insensitive to some local features of the dsDNA. Our simulations demonstrate strong correlated interactions involved in the dsDNA–dsDNA pairing process. Considering monoatomic counterions like Na^+^, K^+^ and Mg^2+^, the sequence-dependent specificity found is of an electrostatic nature, and we expect that non-mean-field interactions play a role in the recognition preference in dsDNA–dsDNA interactions.

## Data Availability

Data available upon reasonable request.

## Supplementary Materials

The following supporting information can be downloaded at: https://www.mdpi.com/article/10.3390/life12050699/s1, Figure S1: Distribution of instantaneous force at chosen separation distances for both flexible and rigid systems; Figure S2: Minimum distance distribution of Na^+^ ions from heavy atoms of dsDNAs in the rigid and flexible structures of Model-180 with the inter-helical distance at 22.4 Å; Figure S3: From left to right are radial distribution of Na^+^ ions number density from the helical center of the dsDNAs, radial distribution of electrostatic potential energy of Na^+^ ions with dsDNAs, a cross section of electrostatic potential energy in rigid structures and flexible structures, respectively; Figure S4: The effective sodium sites in the grooves and interface zone in the rigid and flexible Model180 and Model-72 with the inter-helical distance *d* = 22.4 Å, respectively; Table S1: Probabilities that a location randomly chosen from the flexible system has higher diffusion coefficient than a location randomly chosen from the rigid one.
