# Supplementary material for: The Effects of Flexibility on dsDNA–dsDNA Interactions
Source: Life (Basel). 2022 May 7;12(5):699. doi: 10.3390/life12050699 (PMC9147707; doi:10.3390/life12050699)
Supplement: Supplementary file 1 [file life-12-00699-s001.zip › life-1695554-supplementary.pdf]

Supporting Information

The effects of flexibility on dsDNA-dsDNA interactions

Chuanying Chen, B. Montgomery Pettitt*

Department of Biochemistry & Molecular Biology, Sealy Center for Structural Biology and Molecular Biophysics, University of Texas Medical Branch, Galveston, TX 77555

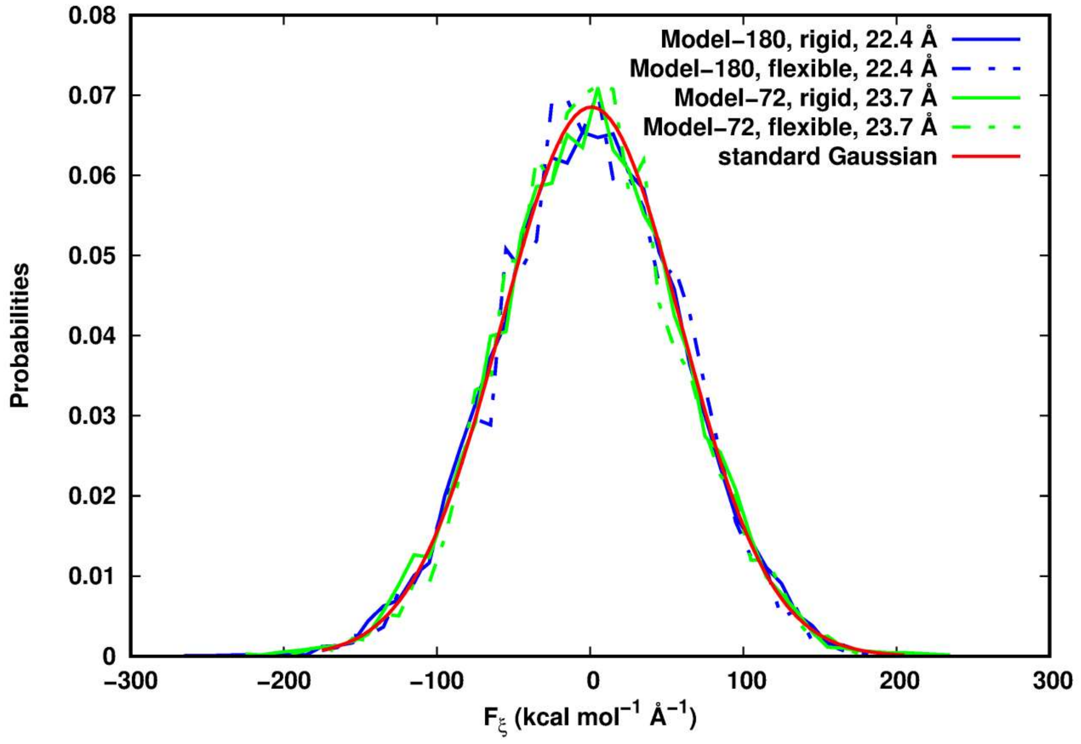

Figure S1. Distribution of instantaneous force at chosen separation distances for both flexible and rigid systems.

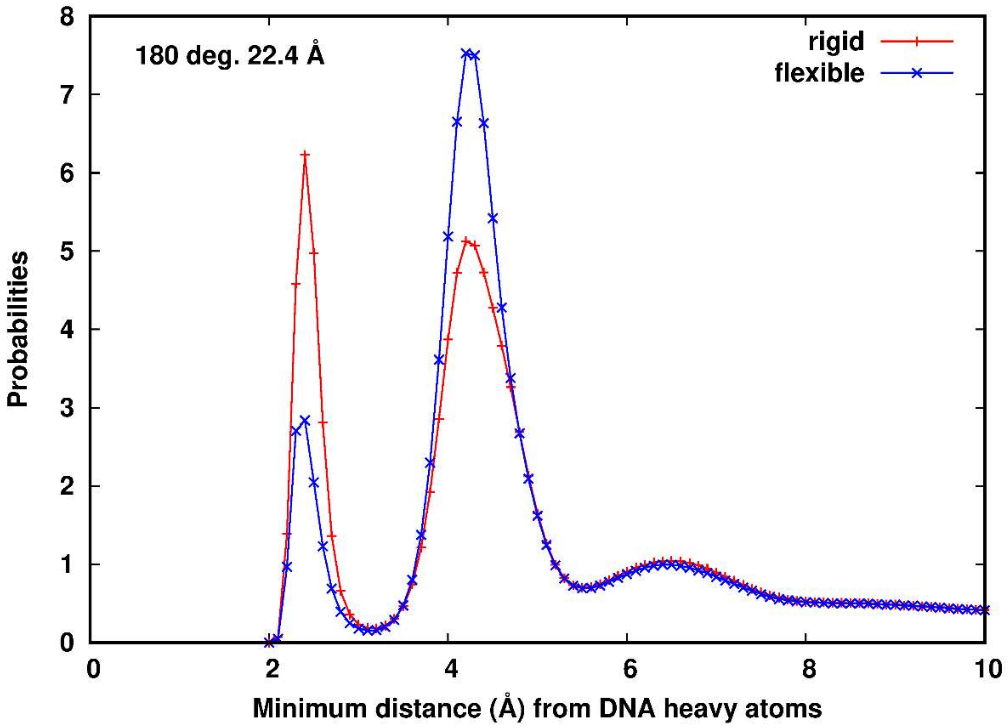

Figure S2. Minimum distance distribution of Na^+ ions from heavy atoms of dsDNAs in the rigid and flexible structures of Model-180 with the inter-helical distance at 22.4 Å. The distributions for the other configurations with different distance separations are similar. A first sharp peak centered at 2.4 Å corresponds to a direct contact of sodium ions to DNA phosphates or base group oxygen atoms, and a second broader peak at ~ 4.2 Å corresponds to the interaction of the hydrated Na^+ ions with the negatively charged atoms.

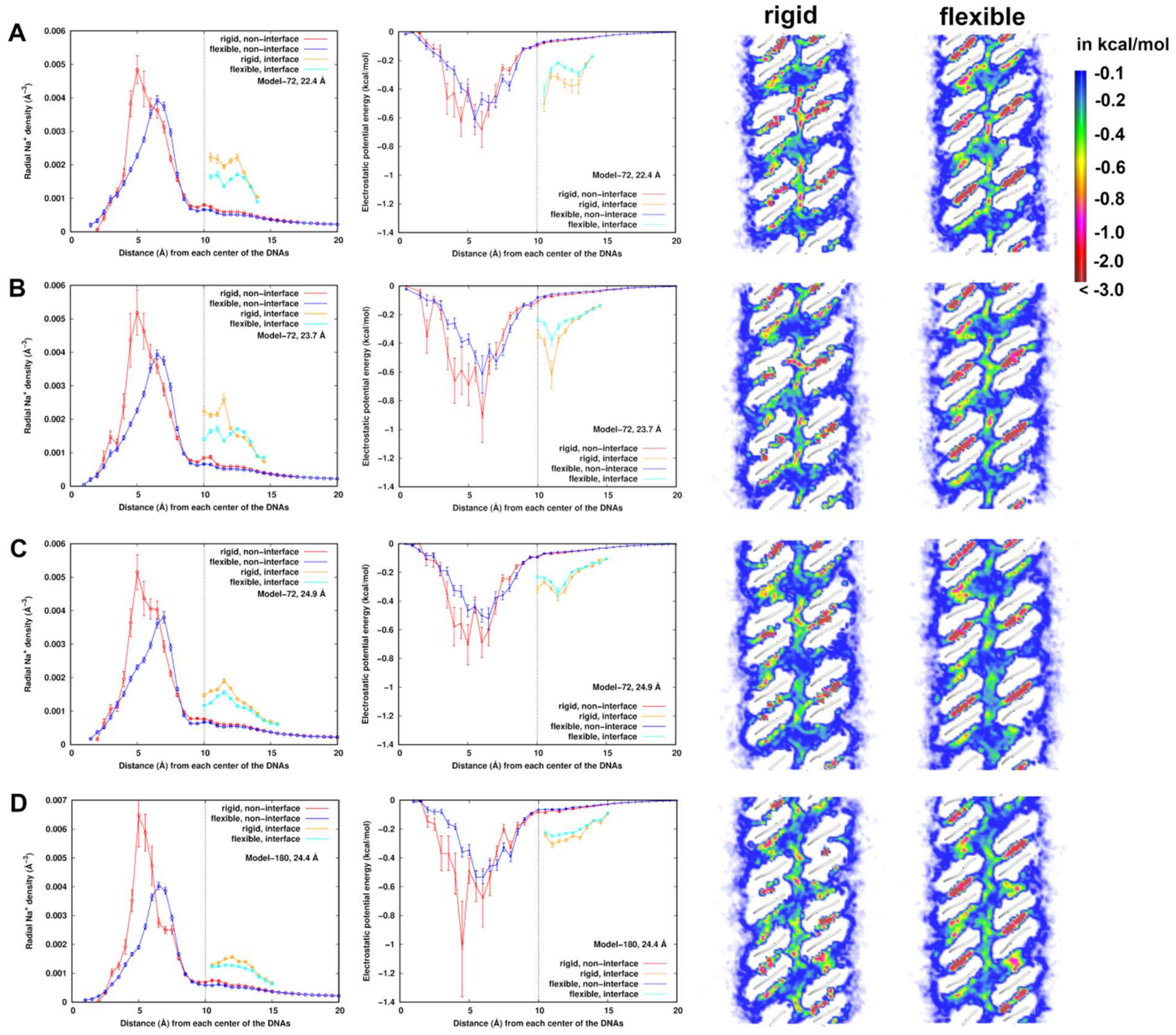

Figure S3. From left to right are radial distribution of Na^+ ions number density from the helical center of the dsDNAs, radial distribution of electrostatic potential energy of Na^+ ions with dsDNAs, a cross section of electrostatic potential energy in rigid structures and flexible structures, respectively. (A) Model-72 with the inter-helical distance d = 22.4 Å; (B) Model-72 with d = 23.7 Å; (C) Model-72 with d = 24.9 Å and (D) Model-180 with d = 24.4 Å. Backbones of dsDNAs are in color of white.

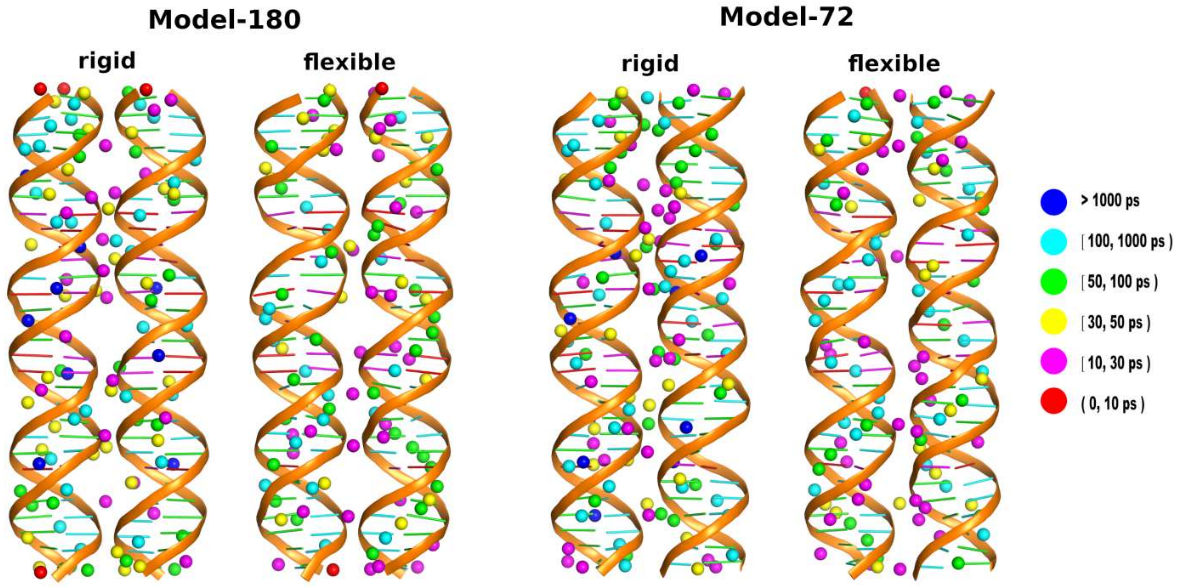

Figure S4. The effective sodium sites in the grooves and interface zone in the rigid and flexible Model180 and Model-72 with the inter-helical distance d = 22.4 Å, respectively. The sites are colored with respect to residence time. The sites were finally identified until the separation of the sites is larger than 2.8 Å. The DNA bases are colored: A, red; T, magenta; G, green; and C, cyan. To identify effective sodium sites in a particular region, we first divided the trajectory into several blocks and generated sodium density map using a grid size of 0.5 Å for each block; then for the grid points whose densities are larger than a threshold (> 0.01 # / point), we extracted the points which are ~70% overlapped; we smoothed the points by density-weighted averaging the 6-closest neighbors.[1] A residence time at a site was approximated as the inverse slope of the linear region in log(C(t)) by linear least-squares fitting, in which C(t) is a survival time correlation function representing the average number of Na^+ ions that remain in a site for a duration of time.[2]

Table S1. Probabilities that a location randomly chosen from the flexible system has higher diffusion coefficient than a location randomly chosen from the rigid one.

	minor groove	major groove	interface
Model-180, 22.4 Å	0.66	0.59	0.61
Model-180, 24.4 Å	0.69	0.59	0.59
Model-72, 22.4 Å	0.67	0.60	0.65
Model-72, 23.7 Å	0.69	0.58	0.61
Model-72, 24.9 Å	0.69	0.57	0.61

References

1. Lai, C. L.; Chen, C.; Ou, S. C.; Prentiss, M.; Pettitt, B. M., Interactions between identical DNA double helices. *Phys Rev E* **2020,** *101* (3-1), 032414.

2. Feig, M.; Pettitt, B. M., Sodium and chlorine ions as part of the DNA solvation shell. *Biophys J* **1999,** *77* (4), 1769-81.
